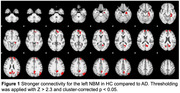# Nucleus Basalis of Meynert: Functional Connectivity and Morphometry in Alzheimer's Disease and Frontotemporal Dementia

**DOI:** 10.1002/alz70856_105620

**Published:** 2026-01-07

**Authors:** Christine Kindler, Grace Gillis, Gaurav V Bhalerao, Jesper L. R. Andersson, Paul McCarthy, Carolin Miklitz, Tony Stoecker, Gabor C Petzold, Ludovica Griffanti, DESCRIBE‐FTD investigators, DELCODE investigators, DANCER investigators; * these authors contributed equally

**Affiliations:** ^1^ Division of Vascular Neurology, Department of Neurology, University Hospital Bonn, Bonn, NRW, Germany; ^2^ German Center for Neurodegenerative Diseases (DZNE), Bonn, NRW, Germany; ^3^ Department of Old Age Psychiatry and Cognitive Disorders, University Hospital Bonn, Bonn, NRW, Germany; ^4^ Oxford Health NHS Foundation Trust, Oxford, Oxfordshire, United Kingdom; ^5^ Department of Psychiatry, Oxford Centre for Human Brain Activity (OHBA), Wellcome Centre for Integrative Neuroimaging, University of Oxford, Oxford, Oxfordshire, United Kingdom; ^6^ Nuffield Department of Clinical Neurosciences, Oxford Centre for Functional MRI of the Brain (FMRIB), Wellcome Centre for Integrative Neuroimaging, University of Oxford, Oxford, Oxfordshire, United Kingdom; ^7^ Department of Physics and Astronomy, University of Bonn, Bonn, NRW, Germany

## Abstract

**Background:**

The nucleus basalis of Meynert (NBM) is crucial for learning, attention, and memory. While its involvement in Alzheimer's disease (AD) has been widely reported, the role in frontotemporal dementia (FTD) remains unclear. Here we examined NBM functional connectivity (FC) as well as NBM and cortical volume changes in AD, healthy controls (HC), and FTD subtypes: behavioral variant FTD (bvFTD), unclassified primary progressive aphasia (PPA), progressive nonfluent aphasia (PNFA), semantic dementia (SemD), and progressive logopenic aphasia (PLA).

**Method:**

Resting‐state fMRI and T1‐weighted scans were collected from HC (*n* = 66), individuals with AD (*n* = 50), bvFTD (*n* = 63), PLA (*n* = 18), PPA (*n* = 20), PNFA (*n* = 32), and SemD (*n* = 15). We performed seed‐based FC analyses in FSL with left and right NBM as seeds. We compared HC with AD (cluster‐based threshold z > 2.3, *p* < 0.05). Significant clusters were used to extract mean FC for the other groups. We then compared FC values and normalized NBM volumes across HC and FTD subtypes using the Kruskal–Wallis test, followed by Bonferroni‐corrected pairwise comparisons where applicable. Voxel‐based morphometry (VBM) was conducted to explore cortical atrophy patterns.

**Result:**

HC showed stronger NBM connectivity than AD in the hippocampus/parahippocampal gyrus, frontal pole, paracingulate cortex, precuneus, and lateral occipital cortex (Figure 1, left NBM results). Across HC and FTD subtypes, we found significant group differences for the paracingulate (H = 36.15, *p* < 0.001) and lateral occipital cortex (H = 18.25, *p* =  0.003). Connectivity was higher in bvFTD, PPA, and LPA than in HC, with the strongest effect for bvFTD in the paracingulate cortex (*r* = 0.48) and moderate effects across other contrasts (*r* = 0.28–0.42, all *p* < 0.020). Volumetric analyses indicated no significant group differences in NBM volumes but distinct cortical atrophy patterns: PNFA, PLA, and PPA exhibited temporal‐ frontal atrophy similar to AD, while bvFTD showed predominantly frontal and SemD primarily temporal atrophy.

**Conclusion:**

Differential functional connectivity of the NBM and distinct cortical atrophy patterns were observed between HC and AD and between HC and FTD subtypes. Ongoing analyses on subgroup comparisons and integration of cognitive assessments aim to elucidate these relationships and their clinical implications.